# Inform and do no harm: Nocebo education reduces false self-diagnosis caused by mental health awareness

**DOI:** 10.1017/S0033291725101979

**Published:** 2025-11-10

**Authors:** Dasha A. Sandra, Zindel Segal, Sanaa Majoo, Anastasia Sistanis, Matthew J. Burke, Michael Inzlicht

**Affiliations:** 1 Department of Clinical Psychological Science, University of Toronto, Toronto, ON, Canada; 2 Faculty of Health Sciences, Ontario Tech University, Oshawa, ON, Canada; 3 Department of Psychology, Toronto Metropolitan University, Toronto, ON, Canada; 4 Neuropsychiatry Program, Department of Psychiatry and Division of Neurology, Department of Medicine, University of Toronto, Toronto, ON, Canada; 5 Hurvitz Brain Sciences Program and Tory Trauma Program, Sunnybrook Research Institute, Toronto, ON, Canada; 6 Department of Psychology, University of Toronto, Toronto, ON, Canada; 7 Rotman School of Management, University of Toronto, Toronto, ON, Canada

**Keywords:** ADHD, expectations, intervention, mental health awareness, Nocebo effect

## Abstract

**Background:**

Mental health awareness efforts are increasing, especially for ADHD. There is growing evidence that such efforts may also cause unnecessary self-diagnosis and worsening symptoms for some disorders; however, there are no validated approaches to avoid these potential harms without reducing the awareness efforts themselves. We developed a multifaceted intervention, called *nocebo education.* The intervention was based on the principles of the nocebo effect, where negative expectations may cause symptom misattribution and worsening. We tested whether teaching about the nocebo effect could mitigate the potential false self-diagnosis and symptom worsening from ADHD awareness.

**Methods:**

In a double-blind randomized controlled trial with a week-long follow-up (NCT06638411), 215 healthy young adults (77% women) were randomized to participate in a group workshop on either ADHD awareness, ADHD combined with nocebo education, or control (sleep). We measured changes in self-diagnosis and ADHD symptoms immediately after the workshop (self-diagnosis), and 1-week later (self-diagnosis and symptoms).

**Results:**

ADHD group reported substantially higher self-diagnosis scores immediately 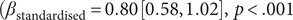 ) and 1 week after the workshop 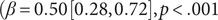 ) compared to controls. These effects persisted despite no changes in reported symptoms. Nocebo education halved the false self-diagnosis scores immediately after the workshop ( 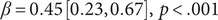 ) and eliminated the false self-diagnosis entirely at follow-up 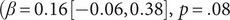 ).

**Conclusions:**

We show that being exposed to ADHD awareness reliably increases false self-diagnosis among healthy young adults for at least one week; a brief nocebo education intervention is efficacious in substantially reducing and later eliminating it. Nocebo education is a promising adjunct for balanced awareness efforts that could be applied in various contexts.

## Introduction

Rates of mental health problems have risen dramatically in North America and worldwide, especially for young adults. Teenagers and young adults report higher rates of mental health problems than any other segment of the population (Askari, Mauro, Kaur, & Keyes, 2023; Botha, Morris, Butterworth, & Glozier, 2023; Twenge, Cooper, Joiner, Duffy, & Binau, 2019; Udupa, Twenge, McAllister, & Joiner, 2023). Over 60% of university students met the criteria for at least one psychiatric disorder in the United States in 2021 (Lipson et al., 2022), with similar rates among youth in Canada (Wiens et al., 2020) and the UK (McCurdy & Murphy, 2024). The concerning rates are exacerbated by the fact that young people are at the highest risk of developing a mental disorder (McGrath et al., 2023; Solmi et al., 2022) – increasing the urgency of what many experts have called a ‘youth mental health crisis’ (Aftab & Druss, 2023; Benton, Boyd, & Njoroge, 2021; Murthy, 2021). In response, various organizations such as universities, NGOs, and mental health advocacy groups ramped up awareness efforts (e.g. in schools; Guzman-Holst, Streckfuss Davis, Andrews, & Foulkes, 2024, on social media; Tam, Wu, Zhang, Pawliuk, & Robillard, 2024) to reduce social stigma (Lucksted & Drapalski, 2015), as well as encourage early detection and treatment. These efforts have had some success: mental health awareness is well known to be associated with reduced stigma and increased help-seeking (Henderson, Robinson, Evans-Lacko, & Thornicroft, 2017).

However, a growing number of studies show that awareness efforts may also cause harms for healthy young adults (Foulkes & Andrews, 2023). These could include false *self-diagnosis*, or the belief one has a disorder without receiving a formal diagnosis, and *iatrogenic symptoms*, or symptoms caused by the very expectation one has a disorder. Several studies show that exposure to awareness about various mental health conditions can lead to false self-diagnosis with them. For instance, during the rise of trauma awareness, one study found that broadening what constitutes a trauma led people to mistakenly identify a mild personal distressing experience as traumatic (Jones & McNally, 2021). More recent studies, coinciding with the rise of anxiety awareness, found that normalizing anxiety disorder online led healthy participants to endorse self-diagnosis at higher numbers (Hasan, Foster, & Cho, 2023). In some cases, such self-diagnosis is accompanied by an associated symptom worsening, but the evidence is mixed. For example, broadening the trauma concept did not lead to increased anxiety (Jones & McNally, 2021), nor did the exposure to normalizing anxiety messaging (Hasan et al., 2023); yet multiple studies found that trigger warnings of distressing content ahead consistently *increased* anticipatory distress rather than reduce it (Bridgland, Jones, & Bellet, 2023). Furthermore, recent preventative school-based interventions and mental health awareness efforts have been documented to reduce well-being or worsen emotional difficulties (Deighton et al., 2025; Guzman-Holst et al., 2024).

Results on other health conditions are similarly mixed. Several studies found that self-diagnosis and presentation of functional Tourette’s-like tics may have increased after learning about them through awareness efforts of health advocates on social media (Fremer et al., 2022; Frey, Black, & Malaty, 2022; Mü Ller-Vahl, Pisarenko, Jakubovski, & Fremer, 2021). Studies on loneliness found that messaging on the dangers of loneliness led to individuals expecting greater harm from their time alone by experiencing greater negative affect and loneliness after solitary time than those with positive beliefs (Rodriguez, Pratt, Bellet, & McNally, 2025; Rodriguez, Schertz, & Kross, 2025). Other studies showed that providing awareness of sham conditions (e.g. electromagnetic frequency sensitivity, food addiction) can cause healthy adults to falsely self-diagnose with them yet show no symptoms for some (Hardman et al., 2015), and report them for others (Bräscher, Schulz, Van Den Bergh, & Witthöft, 2020; Crichton & Petrie, 2015; Verrender, Loughran, Dalecki, Freudenstein, & Croft, 2018; Witthöft & Rubin, 2013). Although the evidence on symptoms is still unclear, awareness efforts can clearly cause false self-diagnosis across a range of conditions. This, in turn, may contribute to the rise in self-reported mental health problems among youth, overdiagnosis, overuse of services (Monteith et al., 2024), and maladaptive coping (Ahuvia, Schleider, Kneeland, Moser, & Schroder, 2024; Foulkes & Andrews, 2023; Haslam & Tse, 2025; Moses, 2009).

### Viable interventions

Experts now consistently call for better approaches to mental health awareness (Fergusson, Reed-Purvis, & Foulkes, 2023; Haslam & Tse, 2025), yet there have been practically no studies testing potential interventions to mitigate unintended harms. While some related interventions could help by reappraising the beliefs about some experiences (e.g. time alone as solitude instead of loneliness; Rodriguez, Bellet, & McNally, 2020; Rodriguez, Pratt, et al., 2025; Rodriguez, Schertz, & Kross, 2025), this method is harder to apply to discrete clinical categories of mental disorders. Reappraisal of symptoms would also require recreating the already existing awareness materials and customizing each awareness message to the specific disorder and to the framing. Instead, we developed a general *nocebo education* (Petrie & Rief, 2019) intervention for a balanced mental health awareness: here, a brief explanation of how negative expectations can lead healthy people to reinterpret normal experiences as signs of a disorder and develop more of these symptoms over time. Our intervention is based on the role of *the nocebo effect*, when negative expectations cause symptom misattribution and worsening, which is well known in medicine (Petrie & Rief, 2019).

Negative expectations consistently increase drug side effects and exacerbate poor health outcomes (Petrie & Rief, 2019). Several contextual factors can contribute to the nocebo effect: learning about side effects from others through social learning (Saunders et al., 2024), developing negative expectations about the likelihood of side effects, as well as misinterpreting normal unrelated experiences (e.g. occasional fatigue, headaches) as relevant symptoms, and then noticing more of these over time (Petrie & Rief, 2019). Mental health awareness efforts may also teach negative expectations by focusing on the stories of people with mild symptoms that are similar to normal experience, and reframing these as disordered (i.e. create ‘concept creep’; Haslam, 2016). This would inadvertently lead to false self-diagnosis and worsening symptoms over time – a converging hypothesis proposed by several experts (Foulkes & Andrews, 2023; Haslam, 2016).

### Current study

Given the parallel between medicine and mental health awareness, our nocebo education intervention included principles similar to those of medical nocebo education. Teaching patients about the role of negative expectations can prevent them from misattributing the symptoms to drug side effects and instead view them as transient – thus experiencing fewer of them (Crichton & Petrie, 2015; Michnevich, Hendi, Clinic, Oechsle, & Stein, 2022; Pan, Kinitz, Stapic, & Nestoriuc, 2019). Therefore, our intervention (Table 1) also included an explanation of the role of negative expectations in causing false self-diagnosis and symptom worsening, in addition to relatable examples for social learning (Saunders et al., 2024), and an opportunity to develop a new *mindset*, or an alternative set of beliefs to change the meaning for one’s distress (Zion & Crum, 2018).

**Table 1.** Components of the multi-faceted nocebo education intervention Participants received the education/expectations, social learning, and normalization components before the mental health workshop as a brief lecture, and the mindset shift component after the workshop as a written reflection exercise

Components	Example of delivery
A trusted authority figure	*Clinical psychologist, physician, teacher*
Education and expectations: How negative expectations can worsen mental health and mood	*Definition of the nocebo effect and its mechanisms* (e.g. misinterpreting random fluctuations as symptoms, paying too much attention to these over time). *Evidence that nocebo symptoms are real* (e.g. data from thousands of clinical trials). *When and where they can occur* (e.g. from reading side effect label for medications, learning about it from a clinician, on the internet, social media, or chatbots).
Social learning: Examples of experiencing the nocebo effect and its successful resolution	*Example:* Hitting your head and being told you have a concussion only to find out later that you do not. *Example:* Having occasional headaches and food cravings but thinking they are side effects of a new antidepressant you just started taking (until you get off antidepressants, but the symptoms stay).
Normalization of experience: Describe some difficulties as normal	
Mindset change: Reframe symptoms from signs of a disorder to potentially normal	*Written exercises or discussion prompts. For example:* Does relating to the symptoms you just learned mean that you may have undiagnosed disorder (e.g. ADHD)? Or could your symptoms have another explanation (e.g. stress, poor sleep, phone distractions, external factors)? How can you use what you learned about the nocebo effect to prevent your expectations from causing symptoms in the future?

In a double-blind pre-registered randomized controlled trial, we tested our nocebo education intervention with young adult university students by inviting them to participate in a mental health awareness workshop for ADHD. We chose ADHD as a condition for two reasons. First, ADHD is somewhat unique: it is both *overdiagnosed* – as a result of rising literacy (Abdelnour, Jansen, & Gold, 2022; Gascon, Gamache, St-Laurent, & Stipanicic, 2022; Kazda et al., 2021), and *underdiagnosed* – due to preferential diagnosis of some (e.g. children, boys) and not other (e.g. adults, women) subsets of the population, thus requiring more awareness efforts (Attoe & Climie, 2023; Faraone et al., 2024; Martin, 2024; Quinn, 2005; Young et al., 2020). Second, ADHD awareness has been increasing rapidly within the broader rise in neurodivergence movement, but ADHD literacy may not yet be as ubiquitous as that of anxiety or trauma. Yet it has already begun to change individual experience: one study found that self-reports of symptom overlap between ADHD and autism is rapidly rising online, despite no changes in base rates in clinical populations (Kang, Haslam, & Conway, 2025). The focus on ADHD thus offers us a window of possibility to 1) confirm the potential nocebo effects of mental health awareness, and 2) test potential interventions that could prevent unintended harms and apply them broadly. We hypothesized that receiving ADHD awareness alone would lead to increases in false self-diagnosis and symptom reporting, but combining it with brief nocebo education would inoculate against these harms, akin to a vaccine inoculation. This study demonstrates the preliminary feasibility and efficacy of a nocebo education intervention to reduce unintended harms of mental health awareness.

## Methods

### Participants

We recruited individuals from a student participant pool and the broader community of the University of Toronto Scarborough. We only recruited participants that scored below 18 on a validated ADHD screener to ensure we excluded only those highly likely to be true cases of ADHD (Kessler et al., 2005). Indeed, although the scale’s cut-off for risk of ADHD is 14, diagnostic accuracy is far higher for those scoring between 18 and 24, rather than 14 to 17. Given that ADHD is well-known to be overdiagnosed (Bruchmüller, Margraf, & Schneider, 2012), we wanted to ensure that we excluded only the true cases of ADHD. Other inclusion criteria were ages 18–25, no prior diagnosis of psychiatric or neurological disorder (including ADHD), no active intake of mental health-related medication (e.g. antidepressants), fluent in English, having access to a computer, tablet, or smartphone with internet, and normal or corrected-to-normal vision and hearing. We screened 499 participants between September and December 2024; 98 did not meet eligibility criteria; 139 could not attend the initial session, 37 had technical issues or did not complete the full study; 10 did not reconsent to data use (see Supplementary Appendix 1). The final sample included 215 participants (77% women, 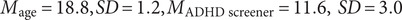 , 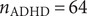 , 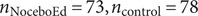 ; Supplementary Appendix 1) from diverse backgrounds.

The total duration of the experiment was approximately 1.5 hours spread over 1 week in three sessions: a 1 hour in-person workshop and two 15-minute online questionnaires at 3 and 7 days after the initial lab visit. Participants received 1.5 course credits or a $15 Amazon gift card for their total participation. We also provided an extra 0.5 credit/$5 incentive to participants to complete all parts of the study. The protocol was approved by the University of Toronto Social Sciences, Humanities & Education Research Ethics Board (#45738) and followed the Declaration of Helsinki.

### Procedure

Prior to the experiment, participants first completed a brief screening survey on Qualtrics to determine their eligibility. They then signed up for the study ostensibly on evaluating the quality of various health workshops, in an effort to improve the quality of messaging from the university’s health center. They were randomly assigned to groups ranging from 4 to 14 based on their schedule availability to participate in the in-person experimental session. This method allowed us to replicate real-world settings, where mental health awareness is often delivered to groups, rather than individually. There was a total of 31 groups 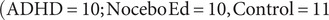 ).

#### In-person initial session

In the beginning of the in-person experimental session, participants met one of two female research assistants (SM or AS) at a classroom in University of Toronto, Scarborough. The research assistant introduced herself as a co-lead of the study and explained the study format and procedure. Participants completed a Qualtrics survey that asked them about their demographic information, baseline self-reported measures of ADHD symptoms (Adult ADHD Self-Report [ASRS] scale), memory failures (Memory Failure Scale [MFS]), affect-based symptoms (Kessler Psychological Distress Scale, K10) and personality measures (Anxiety Sensitivity Index [ASI]) as the T0 measurement time-point. Participants also rated their ADHD self-diagnosis following previously validated self-reported items (Hasan et al., 2023). Participants also responded to two filler questionnaires on sleep quality and self-diagnosis with sleep disorders, to maintain the cover story of the study. After the participants completed the baseline survey, they were block-randomized to one of three conditions: ADHD awareness, control (sleep and dreams), or ADHD + nocebo education group in a 1:1:1 ratio. Groups were randomized by the experimenter prior to data collection, using R (R Core Team, 2021, version 4.1.2).

Once baseline measures and randomization were completed, the research assistant introduced the experimenter (DS) as a clinical psychology PhD student who then delivered the brief 30-minute awareness workshop with condition-specific content. The workshop involved no individual interaction throughout, and participants were discouraged from discussion. In each condition, participants attended a two-part workshop: the first part provided either a generic information on sleep hygiene (for ADHD and control) or nocebo education (ADHD + nocebo education); the second part provided the condition-specific awareness materials.

*ADHD.* This workshop included information sourced from popular ADHD advocacy organizations, and demographic studies on the disorder. It induced *negative expectations* by providing statistics on ADHD in adults, symptom descriptions, and hidden signs of the disorder. The workshop also included *social learning* elements by including a brief video of a well-known ADHD advocate describing her personal experiences with ADHD diagnosis. Finally, participants concluded the workshop with a writing activity, which provided a *mindset and a framework to interpret distress:* they answered questions on the level of acceptance of an ADHD diagnosis, whether they related to any of the symptoms, whether these were indicative of them potentially having ADHD, and how they could manage these symptoms going forward.

*ADHD + Nocebo education.* Participants received a brief 10-minute information session about the nocebo effects before the ADHD content (identical to the ADHD condition). The session included an explanation of the nocebo effect (*inoculating expectations*) and a relatable example of it in daily life (*inoculating social learning*; Michnevich et al., 2022; Quidde et al., 2018). To provide a different *mindset* for interpreting distress, participants completed a modified 5-minute writing activity after the ADHD content portion. Here, they answered some of the same questions from the ADHD condition and then elaborated on the possibility of their symptoms to be due to factors other than underlying ADHD. Participants concluded with proposing strategies to manage their symptoms considering the information they learned.

*Control (sleep).* Participants learned about various unusual sleep experiences such as nightmares, sleep paralysis, and lucid dreaming, as well as the mechanisms of dreaming and the theories explaining their purpose. Participants finished the workshop with a writing activity answering questions on the sleep experiences they have learned about, as well as on the theories of dreaming.

At the end of the workshop, all participants completed a measure of self-diagnosis (T1), as well as a brief questionnaire rating the workshop on various characteristics, in line with the cover story. They then provided their information to receive two follow-up surveys in 3 and 7 days (T2).

#### 3-day follow-up

Here, participants completed a 10-minute online questionnaire where they reported any information remembered from the workshop and any relevant symptoms since attending it. The questionnaire also included filler questions rating the workshop itself.

#### One week follow-up

A week after the workshop, participants completed an online follow-up questionnaire on primary (self-diagnosis, ADHD symptoms, ASRS) and secondary (memory failures, MFS, levels of distress, K10) outcomes. At the end of the survey, participants were assessed for suspicion (Mills, 1976; Nichols & Edlund, 2015). Participants were told that there was an additional element to the study they have not been told about, asked to guess what it is and elaborate their rationale for it and when they started doubting it. We then fully debriefed each participant by providing them with the full description of the true purpose of the study and a brief explanation of the nocebo effect and its mechanisms to remove any belief about potential false self-diagnosis.

#### Blinding

The study was double-blind. Two research assistants led the data collection and were blind to the condition; the experimenter delivering the workshop randomized participants prior to delivering interventions and did not interact with the research assistants or with the participants during any assessments.

### Measures

#### Primary outcomes

*Adult ADHD Self-Report.* The ADHD Self-Report Scale Version 1.1 created by WHO is a self-report screening tool to assess symptoms of ADHD (Kessler et al., 2005). It includes 18 questions to evaluate the frequency of DSM-IV Criterion A symptoms of ADHD, with Part A of the scale containing six items most predictive of ADHD diagnosis (Kessler et al., 2005, 2007); we used this scale due to it being the most popular scale for use in primary care settings. Participants answered questions such as ‘How often do you have trouble wrapping up the final details of a project, once the challenging parts have been done?’ On a five-point scale from 0 (Never) to 4 (Very often). The measure includes a cut-off point of 14 for risk of ADHD and a cut-off of 18 for high risk, with accuracy of an ADHD diagnosis being strongest for the cut-off point of 18 (Kessler et al., 2005, 2007). The screener has a score range of 0–24 and the full measure has a range of 0–72, with a higher score indicating worse symptoms. For the study, we used the Part A screener to determine participant eligibility, and the full scale as measurement of ADHD symptoms. We also evaluated the symptoms over the previous week instead of 6 months, given the duration of the study. The measure showed good reliability in our sample (α = 0.85); subscales showed lower, albeit adequate, reliability: (*α*_inattentiveness_ = 0.79, *α*_impulsivity_ = 0.79; *α*_screener_ = 0.62).

*ADHD self-diagnosis.* To determine participants’ self-diagnosis, they scored one item ‘I believe I have ADHD’ on a scale from 1 (Strongly disagree) to 5 (Strongly agree).

#### Secondary outcomes

*Memory Failures Scale (MFS).* The scale was developed to measure common memory failures in everyday life (Cheyne, Carriere, & Smilek, 2006). Participants rate their experiences on a scale from 1 (Never) to 5 (Very often) for each of the 12 items, for example ‘Even though I put things in a special place, I still forget where they are’. The scale ranges in scores from 12 to 60, with higher scores indicating more failures. The measure had good reliability (*α* = 0.82).

*Kessler Psychological Distress Scale (K10).* This is a brief 10-item screening tool designed to measure non-specific psychological distress (Kessler et al., 2002). Participants rate each item, such as ‘During the last month, about how often did you feel depressed?’ on a scale of 1 (None of the time) to 5 (All of the time). The scale has been validated to use for different time-periods, including for 1 week duration used in the study (Merson, Newby, Shires, Millard, & Mahoney, 2021). The scale score ranges from 10 to 50, with higher scores indicating more symptoms. The measure had strong reliability (*α* = 0.88).

#### Trait level measures

*Anxiety Sensitivity Index.* The ASI is a 16-item scale that measures anxiety sensitivity, or the fear of anxiety-related sensations due to their perceived negative consequences (Reiss, Peterson, Gursky, & McNally, 1986). Participants rate items such as ‘It scares me when I feel shaky’, on a five-point scale ranging from 0 (Very little) to 4 (Very much). The measure had good reliability (*α* = 0.83).

*Self-Concept Clarity (SCC).* The SCC scale is a 12-item questionnaire measuring the stability, consistency, and coherence of one’s self-understanding as well as confidence in one’s self-beliefs. It includes two normally scored items such as ‘In general, I have a clear sense of who I am and what I am’, and 10 reverse-scored items such as ‘My beliefs about myself often conflict with one another’. Participants rate each item on a scale from 1 (Strongly disagree) to 5 (Strongly agree), such that overall higher scores indicate higher self-concept clarity. The measure had good reliability in our sample (*α* = 0.85).

### Statistical analysis plan

All data were analyzed at the individual level. We preregistered all analyses and measures on ClinicalTrials.gov (https://clinicaltrials.gov/study/NCT06638411). We planned to use mixed regressions to predict self-diagnosis and ADHD symptoms given the condition (ADHD, ADHD + nocebo, control) at 1-week follow-up (and immediately for self-diagnosis), with the covariate of baseline symptoms, as well as a random intercept for each participant and cluster to control for possible non-independence of observations within each cluster/workshop group. We also intended to control for the number of participants in each cluster. Overall, we planned to run directional tests and used Type I error rate of 0.05 for all tests. We estimated we needed a sample size of 240 to obtain 80% power to detect small to medium effects (Cohen’s *d* = 0.35).

We had two deviations from this original analysis plan. First, we originally randomized 262 participants, consistent with our pre-registered power analysis of sampling 240 participants after exclusions. However, attrition and exclusion rates were greater than anticipated and resulted in the final sample of 215 participants. Nevertheless, this sample size still allowed for detecting small-to-medium effect sizes (*d* = 0.38) with 80% power. Second, we pre-registered our analyses based on an assumption of normality of data; however, the data on self-reported diagnosis violated that assumption. As a result, we performed robust (i.e. non-parametric) regressions on our data analyses and did non-directional testing for all outcomes; this provided a more conservative, and thus reliable, estimate for our findings.

## Results

### Confirmatory analyses

**Figure 1.**
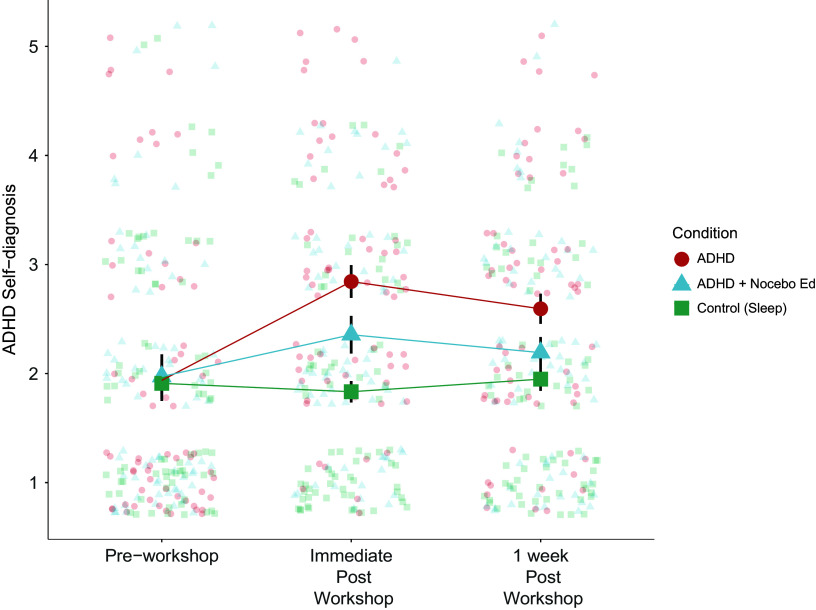
Healthy participants (*N* = 215) reported higher self-diagnosis scores after receiving ADHD awareness workshop when compared with controls. Nocebo education inoculated against these effects partially at immediate post-workshop assessment, and entirely at 1-week follow-up. The graph shows the raw scores on the item ‘I believe I have ADHD’. Large colored dots show means, small colored dots show individual raw scores, and error bars show 95% confidence intervals.

Participants who learned about ADHD reported higher self-diagnosis scores immediately (T1; 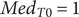 , 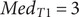 , 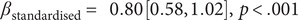 ) and after 1 week (T2; 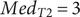 , 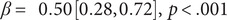 ) compared to controls (Figure 1). The participants’ higher ratings (3–5 out of 5 score of the statement ‘I believe I have ADHD’) for self-diagnosis doubled after the ADHD workshop (28% to 58%, or 30% increase, compared with 29% to 27%, or 2% decrease, in the control group, respectively) at immediate assessment.

Nocebo education mitigated the negative effects of ADHD awareness: participants reported substantially lower increases (half) in self-diagnosis, when compared with those in the ADHD group at both assessment time-points ( 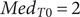 , 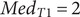 , 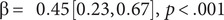 ; 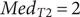 , 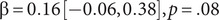 ); participants in this group were indistinguishable from controls at 1-week assessment. Descriptively, the self-diagnosis increase was only half as pronounced in the nocebo education group (26% to 41%, or 15% increase). Furthermore, while in the ADHD group self-diagnosis ratings remained high 1 week later (52%, compared to 30% for controls), only 35% of those in the nocebo education endorsed a higher ADHD self-diagnosis score.

Baseline higher ADHD symptom scores collected during the screening did not predict increases in self-diagnosis scores 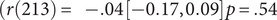 ). Despite the persistent self-diagnosis score increase at 1-week follow-up, we found no changes in overall ADHD symptoms for the ADHD group 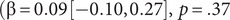 ) or for the nocebo education group ( 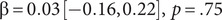 ). The subscale of inattentive symptoms showed reductions in the ADHD group, compared to controls 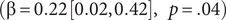 , whereas the subscale of impulsive symptoms showed no changes 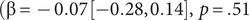 ). However, these reductions are likely artifacts of the study due to the pre-existing group differences at baseline.

To ensure that our findings were robust against participant unblinding, we also tested the sample after excluding those suspicious of the study (*N =* 203). However, we found no differences between the primary outcomes when analyzing data with the 12 suspicious participants (6% of the sample) excluded at T1 ( 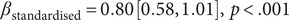 ) and T2 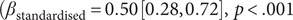 ). The pattern of results for nocebo education group comparison with controls was similar ( 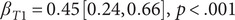 ; 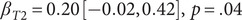 ).

There were also no differences on any of the secondary outcomes of memory (MFS), and distress (K10), or moderating effects of personality measures (ASI and SCC). All analyses were performed at the individual level; we report medians instead of means for self-diagnosis outcomes due to the non-normality of the data.

## Discussion

As mental health awareness continues to increase, it will become progressively more important to provide balanced efforts that can both inform and do no harm. Our brief nocebo education intervention bookending an awareness workshop dramatically reduced and then eliminated unintended self-diagnosis among healthy young adults at a 1-week follow-up. The participants’ higher ratings for self-diagnosis doubled after the ADHD workshop at immediate assessment, yet the increase was only half as pronounced in the nocebo education group. Furthermore, while in the ADHD group self-diagnosis ratings remained similarly high one week later, those in the nocebo education group self-diagnosed at rates similar to those of controls. It is likely that explaining the symptoms and experiences as fluctuations due to previous expectations and normal variability provided enough inoculation to balance the ADHD information. Our intervention was brief (10 minutes) and simple to administer, making it scalable and easy to implement in many settings, for example, schools or universities, where mental health awareness is often given through in-person workshops and lectures.

This study also builds on previous evidence for other mental health conditions and confirms that simply participating in an ADHD awareness workshop can lead to increases in self-diagnosis scores for healthy young adults. Even more importantly, these increases persist for at least a week. Ours is perhaps the largest and the longest-persisting effect in the literature to date on unintended self-diagnosis from mental health awareness. Yet, despite these clear and persistent changes, participants did not report increased symptoms, nor did their past symptoms correlate with the likelihood of endorsing higher self-diagnosis scores after the workshop. This reduces the possibility that individuals with subthreshold symptoms validly identified themselves as having functionally impairing but subclinical symptoms. More broadly, it suggests a disconnect between the individuals’ self-understanding (i.e. self-assessment of having ADHD) and their actual experience (i.e. reported symptoms). Young adulthood is a critical time for developing a stable and coherent identity (Branje, de Moor, Spitzer, & Becht, 2021); being offered a clear label and explanation for otherwise confusing experiences of this period could be particularly seductive but potentially cause long-lasting negative consequences such as maladaptive coping (Ahuvia et al., 2024; Moses, 2009) or formal help-seeking behaviors (Tse & Haslam, 2024).

### Strengths

Our results demonstrate that combining mental health awareness efforts with brief nocebo education can balance increasing mental health literacy while avoiding unintended false self-diagnosis. Such an intervention could be easier and more feasible to implement than fundamentally changing the content of awareness efforts or simply speaking about mental health less. Limiting awareness efforts could risk undoing the progress in reducing stigma, whereas ‘speaking about mental health with more knowledge’ (Morehead, 2025), as some suggest, may require overhauling an innumerable amount of already existing mental health information. Instead, adding nocebo education may be a straightforward adjunct that does not require major changes in existing awareness efforts, yet reduces the associated harm. Given that the intervention is based on general expectations principles (Petrie & Rief, 2019), it could also potentially be adapted to a broader range of contexts, notably online, on social media, or for conversational chatbots.

Beyond the benefits of the intervention, the study design itself also had several strengths. It used careful blinding of participants, which resulted in extremely low rates of suspicion of the true purpose of the study (6% of the sample). Our findings of the efficacy of nocebo education intervention to reduce the false self-diagnosis are thus unlikely to be due to demand characteristics but rather illustrate a real effect of the intervention. Furthermore, we used popular mental health awareness materials that are easily accessible on high-profile websites, making our findings more relevant to real-world settings. Finally, we recruited healthy young adult participants that did not meet the clinical threshold for ADHD symptoms according to a widely used clinical screener (ASRS), were never diagnosed with ADHD, and did not report having a diagnosis of any other mental disorder. Thus, we likely tested the healthiest subset of the relevant population targeted for mental health awareness; individuals with prior diagnoses of other disorders, co-morbid physical health conditions, or a mix of undiagnosed clinical and sub-clinical symptoms may be at even more risk of false self-diagnosis or self-misdiagnosis than identified here.

### Limitations

Our study also had some limitations. For instance, we did not measure help-seeking intentions or behaviors in our participants after the workshop. Without such measures, the full extent of practical benefits of the inoculating intervention is harder to determine and would require further study. Additionally, given that our intervention included several components (i.e. explanation of expectations, social learning, mindset induction), the precise mechanism or the relative efficacy of each is unclear. For this intervention, we chose a ‘shotgun’ approach proposed in previous studies, where we first determined whether the intervention itself is feasible and efficacious, before assessing the specific mechanisms and essential components (Olson, Lifshitz, Raz, & Veissière, 2021; Olson, Sandra, Chmoulevitch, Raz, & Veissière, 2023). Finally, we did not find evidence for symptom worsening in the ADHD condition. We used a modified measure of ADHD symptoms (changing it from the timeframe of 6 months to 7 days), which may have affected its ability to detect changes. Thus, we limit our conclusions on changes in symptoms.

### Future research and implications

Future studies could explore the scalability of our intervention by adapting it to online contexts, such as social media and conversational chatbots. Most of the awareness efforts now take place online, with more than 20% of people now using ChatGPT for health information and advice (Yun & Bickmore, 2025). This adds unique risks of increasing rates of false or inaccurate self-diagnosis (Alho et al., 2024; Rosenquist, Fowler, & Christakis, 2011). Studies could test whether our intervention can effectively inoculate from harms of consuming mental health awareness in these contexts, either as a one-shot intervention (similar to the in-person format of a workshop) or as repeated exposures. Additionally, studies could test the role of the timing of the intervention. Researchers could also track practical implications of false self-diagnosis and inoculation against it, such as changes in intentions to seek help or actual help-seeking behavior (e.g. formal diagnosis, treatment, or school/university accommodations). Extending these findings could help develop optimal approaches to balance mental health awareness benefits and maximally reduce its harms.

Our findings may be immediately useful to school and university administrators, as well as mental health organizations providing awareness efforts. For example, preventative school-based interventions are widely implemented in schools in certain countries such as the UK (Foulkes & Andrews, 2023; Guzman-Holst et al., 2024), while mental health awareness workshops are popular in the US and Canada. Incorporating nocebo education into these interventions could plausibly reduce some of their harms without substantially altering the overall curriculum. For example, practitioners could incorporate nocebo education into the early modules (given a multi-week structure of school-based interventions) and remind students of it throughout subsequent modules. Workshops could adapt nocebo education even more easily by simply bookending the existing workshops with the intervention, similar to the procedure in this study.

More broadly, our intervention could provide some much-needed clarity, or at least nuance, in inherently unclear and confusing conversations about mental health. Mental disorders are difficult to diagnose due to the very nature of diagnostic criteria (Hyman, 2010). Efforts often veer into one of the extremes of either highlighting any negative symptoms as a potential illness (Haslam & Tse, 2025) or of minimizing any distress (Morehead, 2025). As a result, ‘efforts to raise mental health awareness end up raising mental health anxiety just as much’ and lead to false self-diagnosis and panic about mental health rather than pro-active steps or better understanding (Morehead, 2025). Our intervention, instead, provides the awareness of what clinical distress looks like, all the while highlighting that some discomfort is a normal and unescapable part of life.

## Supporting information

Sandra et al. supplementary material Sandra et al. supplementary material

## Supplementary material

The supplementary material for this article can be found at http://doi.org/10.1017/S0033291725101979.

**Additional analyses:** In the spirit of completeness, we also determined whether the missing data from incomplete participation altered the findings via imputation (N = 242). The results were similar for ADHD (  ),  ), and nocebo education group comparisons  ).
