# Supplementary material for: Inform and do no harm: Nocebo education reduces false self-diagnosis caused by mental health awareness
Source: Psychol Med. 2025 Nov 10;55:e330. doi: 10.1017/S0033291725101979 (PMC13054899; doi:10.1017/S0033291725101979)
Supplement: Sandra et al. supplementary material [file S0033291725101979sup001.docx]

**Appendix 1**

**Table 1.** Participant demographic characteristics.

| *Demographic characteristic* | *Participants* | | |
| --- | --- | --- | --- |
|  | *ADHD awareness (n = 64)* | *ADHD + Nocebo education*  *(n = 73)* | *Control*  *(sleep; n = 78)* |
| *Age, mean (SD)* | *18.9 (1.4)* | *18.6 (1.0)* | *18.9 (1.2)* |
| *Sex* |  |  |  |
| *Male* | *18* | *16* | *15* |
| *Female* | *46* | *57* | *63* |
| *ASRS screener score* | *12.0 (3.0)* | *11.3 (2.9)* | *11.5 (3.0)* |

**

**Figure 1.** Recruitment flow for the randomised controlled trial.
